# Poncirin attenuates CCL4-induced liver injury through inhibition of oxidative stress and inflammatory cytokines in mice

**DOI:** 10.1186/s12906-020-02906-7

**Published:** 2020-04-19

**Authors:** Hadayat Ullah, Ashrafullah Khan, Muhammad Waleed Baig, Naseem Ullah, Naveed Ahmed, Muhammad Khalid Tipu, Hussain Ali, Salman Khan

**Affiliations:** Department of Pharmacy, Faculty of Biological Sciences, Quaid-i-Azam University, Islamabad, Pakistan

**Keywords:** CCL4, Poncirin, Oxidative stress, Anti-oxidant enzymes, Cytokines

## Abstract

**Background:**

In the present study, the poncirin which is flavonoid-7-o-glycosides (isolated from the *Poncirus trifoliata*) in nature was evaluated against the Carbon tetra chloride (CCL4)-induced liver injury. The poncirin have been reported for various anti-inflammatory, analgesic activity etc. Based on the previous studies it was anticipated that the poncirin will ameliorate CCL4-induced liver injury.

**Methods:**

The CCL4-induced acute and chronic liver injury model (albino BALB/c mice) was used. Following the induction of the liver injury various parameters such as food and water intake, body weight and weight to dry ratio changes were assessed. Furthermore, various hematological, biochemical parameters and histological studies such as hemotoxylin and eosin (H and E) staining were performed. The poncirin treatment was also evaluated against the pro-inflammatory cytokines such as interleukin-1β (IL-1β), interleukin-6 (IL-6) and tumor necrosis factor-α (TNF-α) using enzyme link immunosorbant assay (ELISA). The Swiss Target prediction software was used to investigate interaction of the poncirin on the various hepatic enzymes.

**Results:**

The poncirin treatment markedly improved the behavioral parameters such as food and water intake. The liver weight variation was attenuated and total body was improved markedly. The hematological and biochemical parameters were significantly improved compared to the CCL4 treated groups. The anti-oxidants were induced, while oxidative stress markers were reduced promisingly. The H and E staining showed that poncirin treatment significantly improved the histology of liver compared to the CCL4 treated group. Furthermore, the poncirin treatment also evidently decreased the inflammatory mediators.

**Conclusions:**

The poncirin treatment showed marked improvement in behavioral, biochemical and histological parameters following CCL4-induced liver injury. Additionally, the poncirin treatment also markedly improved the antioxidant enzymes, attenuated the oxidative stress markers and inflammatory cytokines.

## Background

Liver is considered as vital organ of the body, which critically regulates several important functions of the body including biotransformation and detoxification of the endogenous and exogenous substances [1]. The liver tends to counter the oxidative stress by inducing the antioxidants mechanism, which neutralizes the reactive oxygen and nitrogen free (RONS) radicals [2, 3]. The liver is armed with the endogenous system of antioxidants enzymes such as glutathione-s-transferase (GST), GSH, Catalase and sulphuroxide dismutase (SOD). These antioxidants system are induced by the hepatocytes to counter the offending oxidative stress [3]. However, when the oxidative stress is increased beyond the neutralizing capacity, the liver is the most vulnerable site for the free radicals related tissue damage [3, 4]. The most commonly implicated oxidative free radicals includes superoxides and peroxides, which are released by various endogenous and exogenous substances. These free radicals leads to the oxidation of the macromolecules such as cell membrane, lipids, proteins and DNA [4]. The oxidative stress mediated liver damage results in clinical hepatitis, fibrosis of the liver, cirrhosis and eventually hepatocellular carcinoma [2]. The oxidative stress is associated with the necrosis and inflammation of the hepatic tissue. The oxidative stress can impart damage to the both parenchymal and non-parenchymal cells but exhibit different responses to the oxidative stress. The parenchymal cells of the liver comprises of 70–80% and cover the entire liver [5]. The oxidative stress alter the parenchymal, extracellular matrix composition and immune cells responses [6]. During oxidative stress damage the various inflammatory mediators which are released includes IL-1β, IL-6 and TNF-α. These mediators tends to further escalate the liver damage by the feedback mechanism [7–9].

The NO is an important physiological as well as pathological mediators and have been implicated hepatitis. The NO is released during the inflammatory process by the iNOS and its induction is facilitated by the various molecular signaling mechanism such as NF-κB and MAPKs [8, 10]. The NF-κB and MAPKs signaling cascades are activated by the upstream signaling molecules such as TIRAF, IRAK and MyD88 [7]. Following stimulation, these upstream proteins tends to induce the activation of the downstream transcriptional factors and concerned genes associated with the inflammation [7]. These signaling proteins are transferred to the nucleus and induce the transcription of the concerned genes. The activation of the concerned genes leads to the production and release of numerous pro-inflammatory mediators [11, 12]. The various etiological factors associated with the liver injury includes viral infection, toxins, drugs and chemicals. Several classes of drugs are in clinical practice to deal with the liver injury depending on the stimulus which trigger the inflammation, however, no encouraging breakthrough has been achieved yet. The researchers are trying to develop effective and potent therapies. The natural products are currently being focused by numerous researchers for the treatment of various debilitating diseases including liver dysfunction and exhibited promising protection in various in-vivo and in-vitro models [6, 13]. In the current study, the poncirin (flavonoid-7-o-glycosides in nature and isolated from the *Poncirus trifoliata*) have been investigated against the CCL4-induced liver injury model in mice. *Poncirus trifoliata* belongs to the genus citrus and the dried immature fruit are used to isolate the poncirin. The poncirin have been reported for various biological activities including inflammation, pain, cancer and rheumatoid arthritis [6, 13]. Based on the previous reports, it was anticipated that the poncirin will confer protection against the CCL4-induced liver injury.

## Methods

### Chemical and reagents

Poncirin was provided by the Professor Yeong Shik Kim (Seoul National University, Republic of South Korea), which was isolated from the *Poncirus trifoliata*. The voucher specimen was deposited at College of Pharmacy, Seoul National University and this project was the continuation of the previous project. The various other chemicals and reagents used in the current study includes silymarin, CCL4, ketamine and xylazine obtained from Sigma Aldrich (Sigma Aldrich, USA). For the quantification of the inflammatory cytokines ELISA kits (Thermo Fisher Scientific, USA) were used.

### Plant materials

**Fig. 1:**
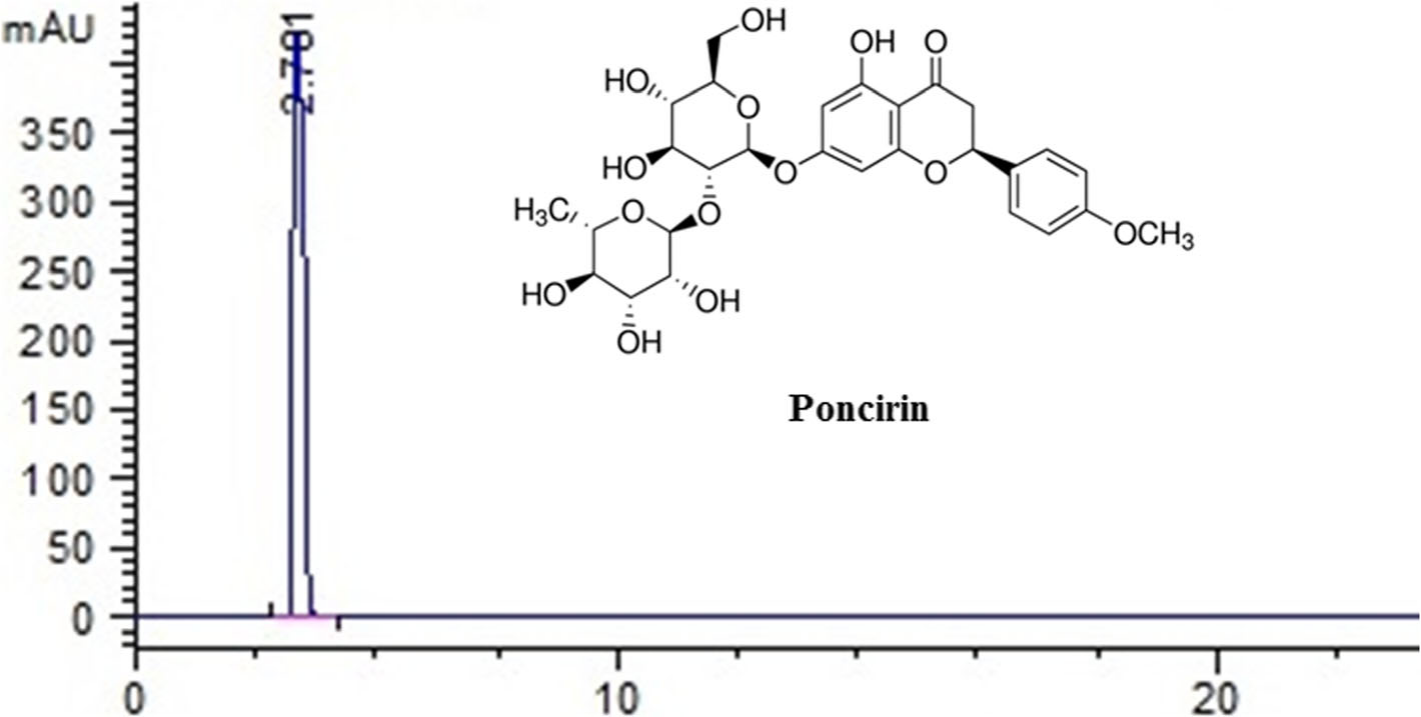
Chemical structure and purity of poncirin

The HPLC analysis was performed to assess the purity as reported previously with necessary modification using Agilent HPLC-DAD (Agilent 1200 series, dual loop autosampler) [14, 15]. The gradient mobile phase comprises of methanol 60% (solvent A) and 40% acid water (solvent B, 1% acetic acid) was used for the analysis, while the flow rate was maintained at 1 ml/sec as reported previously Fig. 1 [14, 15].

### Animals

Male albino mice (BALB/c) were procured during current study, having age of 5–6 weeks and weighing 28–34 g from the NIH, Islamabad, Pakistan. All the animals experiments were performed in the Quaid-i-Azam University Islamabad, Pakistan (Department of Pharmacy, Faculty of biological sciences) pathogen free area (BEC-FBS-QAU2018–90) according to the guidelines issued for the experimental purposes by Quaid-i-Azam University, Islamabad,. The temperature was maintained at 23 ± 0.5 °C, with 12 h light/dark cycle and free excess to water and food [16, 17]. All the animals used in the present study were of healthy nature and no abnormalities/pathology was observed [16, 17].

All the animals were randomly and double blindly assigned to six groups in case of acute study (*n* = 8) as described below.

1. Normal control (normal saline dissolved in 2% DMSO).
2. Negative control (administered with CCL4 2 ml/kg, i.p).
3. Positive control (silymarin 50 mg/kg. i.p).
4. Poncirin treatment (administered at the dose of 5 mg/kg).
5. Poncirin treatment (administered at the dose of 15 mg/kg).
6. Poncirin treatment (administered at the dose of 30 mg/kg).

While in case of chronic study the animals were divided into 4 groups.

1. Normal control (normal saline dissolved in 2% DMSO).
2. Negative control (administered with CCL4 2 ml/kg, i.p).
3. Positive control (silymarin 50 mg/kg, i.p).
4. Treatment control (poncirin was administered at the dose of 30 mg/kg, i.p).

### Acute toxicity assessment

The acute poncirin toxicity was performed in animals as reported previously with slight modification [18, 19]. The animals were divided into five groups (*n* = 5) and poncirin was administered at the dose of 30, 100, 500, 1000 and 1500 mg/kg. Before, the administration of poncirin the animal were fasted overnight. Following administration of the poncirin, the animals were observed for the first 10 h closely for any sign of toxicity. However, for mortality assessment the animals were observed for 24 h [18–20].

### Body weight and liver weight variation analysis

Several disease conditions are associated with the changes in body weight including liver injury. The effect of the CCL4 administration on the body weight changes. The animals of all the recruited groups were weighted before and each day following CCL4-induced liver injury [12]. Similarly, the liver weight of all the treated groups were assessed at the end of the experiments. The mice were euthanized in CO_2_ chamber, liver were separated and weighted [12, 21].

### Water and food assessment

The water and food assessment test was initiated to observe the effect of the poncirin treatment on the CCL4-induced liver injury [12]. The water and food consumption was measured for 24 h in case of acute study, while in case of chronic study the water and food intake assessed each day for 7 days following CCL4-induction in all the treated groups.

### Effect of poncirin on coordination and motor functions

The Kondziela’s tests (inverted mesh scree and weight lifting tests) were performed to evaluate the effect of the poncirin on the CCL4-induced liver injury [22]. The motor coordination and muscle strength assay was commenced on day 0, 1 and 7 after the CCL4 administration [22]. The screen test was performed by placing the mice on the center of the mesh screen and was inverted to record the time of animal holding with the screen as reported. Similarly, the weight test was performed for motor deficit by assessing the animal ability of weight lifting in decreasing order (the weight weights were 20, 27, 33, 46, 59, 72, 85 and 98 g) as reported previously [22].

### Survival rate

The effect of the poncirin treatment on the percent survival rate was assessed in all the treated groups following CCL4-induced liver injury for 7 days [23]. For the survival analysis the animals were randomly assigned to the 4 groups such as normal control, CCL4 control, positive control and treatment control.

### NO determination

The NO is important indicator of the ongoing inflammatory process and can be correlated with extent of inflammation [24]. The effect of the poncirin treatment on the NO production using Griess reagent was evaluated in both tissue and plasma [25]. For the NO determination the blood was directly withdrawn from the heart tissue under deep anesthesia with ketamine and xylazine (60 + 16 mg, i.p). The collected blood was centrifuged at 5000 rpm to separate the plasma from the cellular component and the NO production in plasma was determined as reported with necessary modification [25].

### Hematological and serum analysis

The complete blood analysis was performed to investigate the effect of poncirin treatment on the CCL4-induced liver injury and subsequent alteration of blood composition as reported previously [26, 27]. The serum electrolyte analysis was performed for all the treated groups following CCL4-inudced liver injury and the effect of the poncirin was elucidated as reported before [26, 28]. The effect of poncirin on the Alanine aminotransferase (ALT), Aspartate aminotransferase (AST), Alkaline phosphatase (ALP), total protein and bilirubin analysis was performed for all the treated groups as reported previously [29].

### Histopathological study of liver tissues

The effect of poncirin treatment was evaluated on the CCL4-induced liver injury using hemotoxylin and eosin (H and E) staining [4, 30]. For the commencement of the H and E staining, the liver tissue was isolated, embedded in the paraffin, fixed and dehydrated with the alcohol. The paraffin embedded sample were sectioned in the 3-4 μm, deparrafinised, rehydrated and the stained with the H and E stain [4, 30]. The histology images were assessed under light microscope with 10x magnification and were quantified using Image j software 1.8_172 (NIH, USA) as reported [20].

### Effect of poncirin on liver anti-oxidants and oxidative stress markers

The effect of the poncirin on the anti-oxidants enzymes were studied following induction of liver injury with the CCL4 administration [31]. The CCL4 administration is well toxicant and associated with the severe alteration of anti-oxidant defense system [32]. Furthermore, the CCL4 is related with the production of the oxidative stress and damage of the lipids contents such as lipid peroxidation. The oxidative stress markers such as malonaldehde (MDA) can be used to assess the degree of lipid peroxidation [32, 33]. In short, the effect of the poncirin treatment on the GST, GSH, Catalase and SOD was evaluated against the CCL4-induced liver injury. For the determination of the GST, 0.1 ml of the liver homogenate was mixed with the equal volume of 1-chloro-2,4-dinitrobenzene (CDNB) and the final volume was adjusted to 3 ml by the addition of the phosphate buffer (PH: 6.0) [34]. The absorbance was recorded at 314 nm. The GSH concentration was determined by mixing of 0.1 ml liver homogenate with the 0.5 ml of the 5,5-dithio-bis-2-nitrobenzoic acid (DTNB) and the final volume was made 3 ml by the addition of the phosphate buffer, while the absorbance was recorded at 412 nm [34]. The Catalase concentration was assessed in liver homogenate by adding 0.1 ml of the tissue homogenate with the 2.9 ml of H_2_O_2_ buffer and the absorbance was recorded at 240 nm. The SOD concentration was determined in the liver homogenate by the mixing the 0.2 ml of the sample with the 2.6 ml of Tris-EDTA and 0.1 ml of the pyragallol, while the absorbance was noted at 420 nm [34]. However, the lipid peroxidation product i.e. MDA was determined by incubation of the 0.25 ml tissue homogenate at 37 °C for 1 h in water bath. After this, 5% of the trichloroacetic acid (TCA) and 0.5 ml (0.67%) thiobarbituric acid (TBA) was added to the tissue homogenate and the reading was noted at 535 nm [34].

### Effect of the poncirin on myeloperoxidase (MPO) activity

The myeloperoxidase enzyme commonly used to assess the neutrophilic infiltration into the site of inflammation [35, 36]. The MPO assay was performed using Hexadecyltrimethylammonium bromide (HTAB) and o-dianisidine as reported previously [35, 36]. The tissue were treated and homogenized in the HTAB in 50 mM phosphate buffer to lyse the cells and release the MPO. The HTAB treated tissue were freezed and thawed thrice and subjected to the centrifugation. The HTAB treated tissue supernatant was mixed with the o-dianisidine and H_2_O_2_. The absorbance was reported at 460 nm and the assay performed in triplicate [35, 36].

### Evans blue vascular permeability

The permeability of Evans blue into the tissue such as peritoneum and liver were assessed in all the treated groups according to the reported methods with little modifications [34]. The vascular permeability was assessed by administering the Evans blue dye 30 min after the poncirin administration into the tail vein. Following Evans blue administration, all the animals were injected 0.6% of the acetic acid (i.p). Fifty min after the acetic acid injection, the animals were killed by the cervical dislocation and the fluids was collected from the peritoneum by injecting up to 8 ml of the normal saline as reported [34]. The permeability of the Evans blue dye into the tissue was determined using UV-VIS spectroscopy at 610 nm [34].

### Measurement of IL-1β, IL-6 and TNF-α production

The cytokines are key players of inflammatory process and the increase production of these mediators are associated with worse prognosis of the inflammatory disease [5]. The most commonly implicated inflammatory cytokines in various diseases includes IL-1β, IL-6 and TNF-α [5]. The effect of poncirin was evaluated against the CCL4-induced pro-inflammatory cytokines productions. The tissue samples from all the recruited groups such as normal control, CCL4 treated, positive control and poncirin treated group were removed and the proteins were extracted to determine the concentration of the IL-1β, IL-6 and TNF-α. For this purpose 100 mg of the liver tissue/ml was treated with the PBS containing 0.4 M NaCl, Tween 20 (0.05%) and protease inhibitors were added. Then the samples were homogenized and centrifuged at 3000 g for 10 min to obtain the supernatant for the quantification of the inflammatory cytokines. The IL-1β, IL-6 and TNF-α were determined using commercially available ELISA kits (eBioscience, Inc., San Diego, CA) [5].

### Molecular docking study

The molecular docking study was performed to assess the interaction of the poncirin with the various molecular targets. The Swiss Target Prediction version-2019 was used to study the interaction of the poncirin with the cytochrome p450, adenosine receptor A1, sodium/glucose transporter1/2, low affinity sodium/glucose cotransporter [37].

### Statistical analysis

For the statistical analysis one way analysis of variance (ANOVA) was used followed by the Dunnet’s t test (SPSS, Version 20.0). Data was reported as mean ± standard deviation. *P* value of < 0.05 was chosen for the statistical significance from control group.

## Results

### Effect of the poncirin on the acute toxicity

The poncirin did not exhibited any mortality up to the dose of 1500 mg/kg body weight and no animal mortality was observed in all the recruited groups. However, at the dose of 1500 mg/kg animals showed sign of weakness and sluggish movement as shown in Fig. 6.

### Effect of poncirin treatment on the body weight and liver weight

**Fig. 2:**
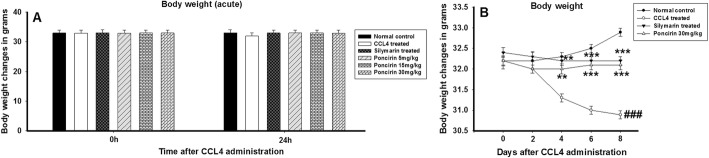
The effect of the poncirin treatment on the CCL4-induced weight changes in both acute (**a**) and chronic (**b**) model in liver injury model. The CCL4 treatment did not alter the body weight during acute study, however, during chronic study significant change in the CCL4 treated animals were noticed. However, the poncirin and silymarin treated group animals showed marked improvement in the body weight when compared to the CCL4 treated group. The data was reported as mean ± standard deviation (*) *p* < 0.05, (**) *p* < 0.01, (***) *p* < 0.001, and (###) shows significant difference comparison with CCL4 treated group

**Fig. 3:**
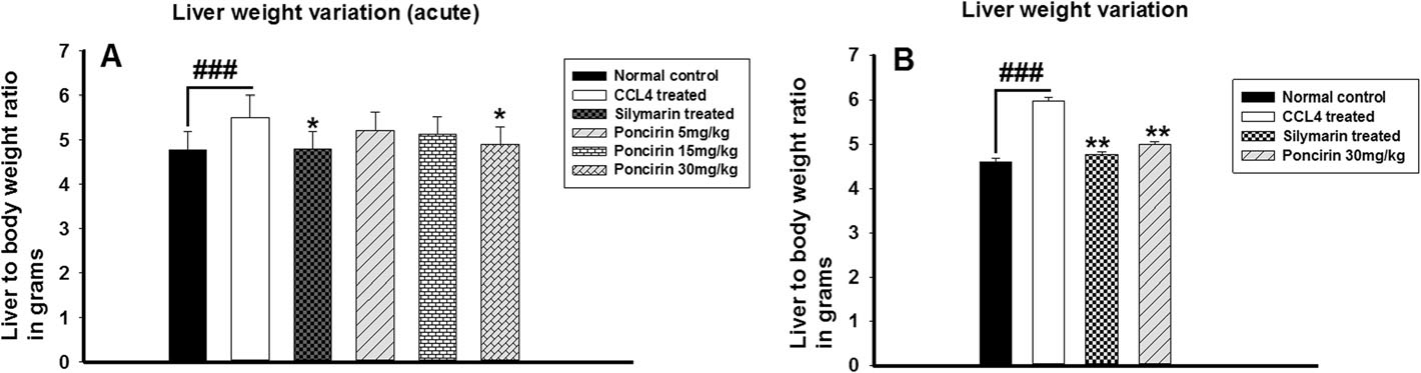
The effect of poncirin treatment on the liver weight variation both acute (**a**) and chronic (**b**) in CCL4-induced liver injury model. The CCL4 treated group showed mild increase in the liver weight variation in acute model (**a**), however, marginal increase in the liver weight variation was observed in case of chronic model. The poncirin treatment markedly attenuated the liver weight variation ratio compared to the CCL4 treated group. The data was reported as mean ± standard deviation (*) *p* < 0.05, (**) *p* < 0.01, (***) *p* < 0.001, and (###) shows significant difference comparison with CCL4 treated group

The body weight and liver changes were determined to assess the impact of the poncirin treatment on the overall changes in body weight and liver weight following liver injury induction with the CCL4. The general body weights were assessed for the 7 days, while the liver weight were measured at the end of the experiment. The poncirin treatment markedly improved the body weight in contrast to the negative control Fig. 2. Similarly, following injection of the CCl_4_, significant increase in the liver weight was observed, however, poncirin treatment decreased the liver inflammation and hence, subsequently liver weight. The liver weight variation were quantified using formula i.e. liver weight/body weight*100 Fig. 3.

### Effect of poncirin treatment on water and food assessment following CCL4-induced liver injury

**Fig. 4:**
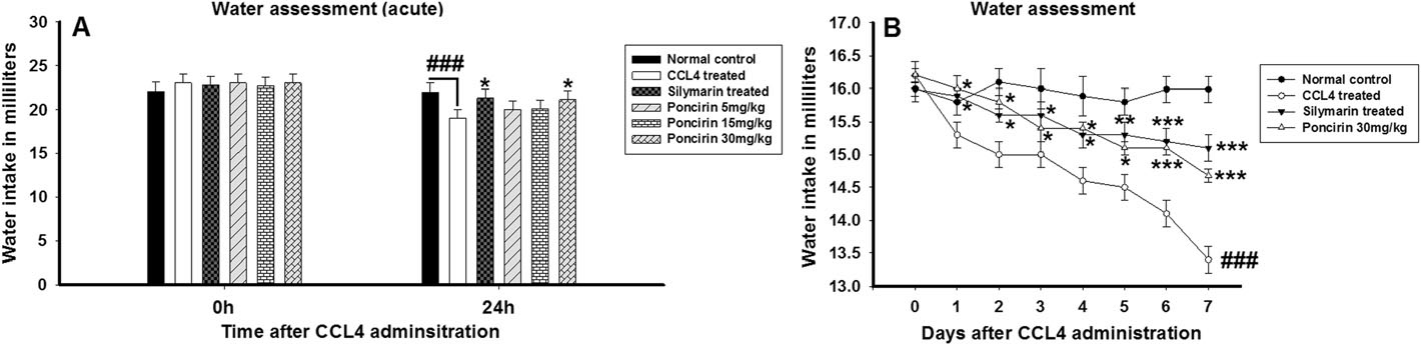
The effect of the poncirin treatment on water intake in both acute (**a**) and chronic (**b**) CCL4-induced liver injury. The poncirin treatment significantly improved the water intake in both acute and chronic model compared to the CCL4 treated group. Similarly, the silymairn treatment also improved the water intake compared to the CCL4 treated group. The data was reported as mean ± standard deviation (*) *p* < 0.05, (**) *p* < 0.01, (***) *p* < 0.001, and (###) shows significant difference comparison with CCL4 treated group

**Fig. 5:**
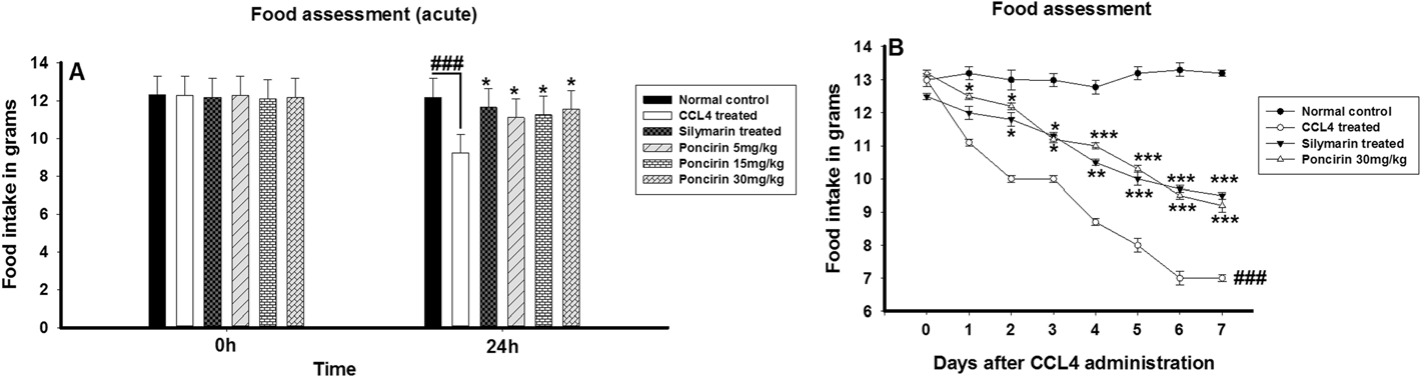
The effect of poncirin treatment on the both acute (**a**) and chronic (**b**) food intake in CCL4-induced liver injury model. The CCL4 administration reduced the food intake in both acute and chronic model. However, the poncirin and silymarin treated group showed marked improvement in the food intake compared to the CCL4 treated group. The data was reported as mean ± standard deviation (*) *p* < 0.05, (**) *p* < 0.01, (***) *p* < 0.001, and (###) shows significant difference comparison with CCL4 treated group

The water and food intake was determined in all the treated groups such as normal control, negative control, positive control and poncirin daily for 7 days. The CCL4 treated groups showed marked decrease in the water and food consumption. The positive control and poncirin treated group showed significant improvement in the water intake and feeding behavior as evident from the Figs. 4 and 5.

### Effect of poncirin treatment on the CCL4-induced muscle strength and coordination

**Fig. 6:**
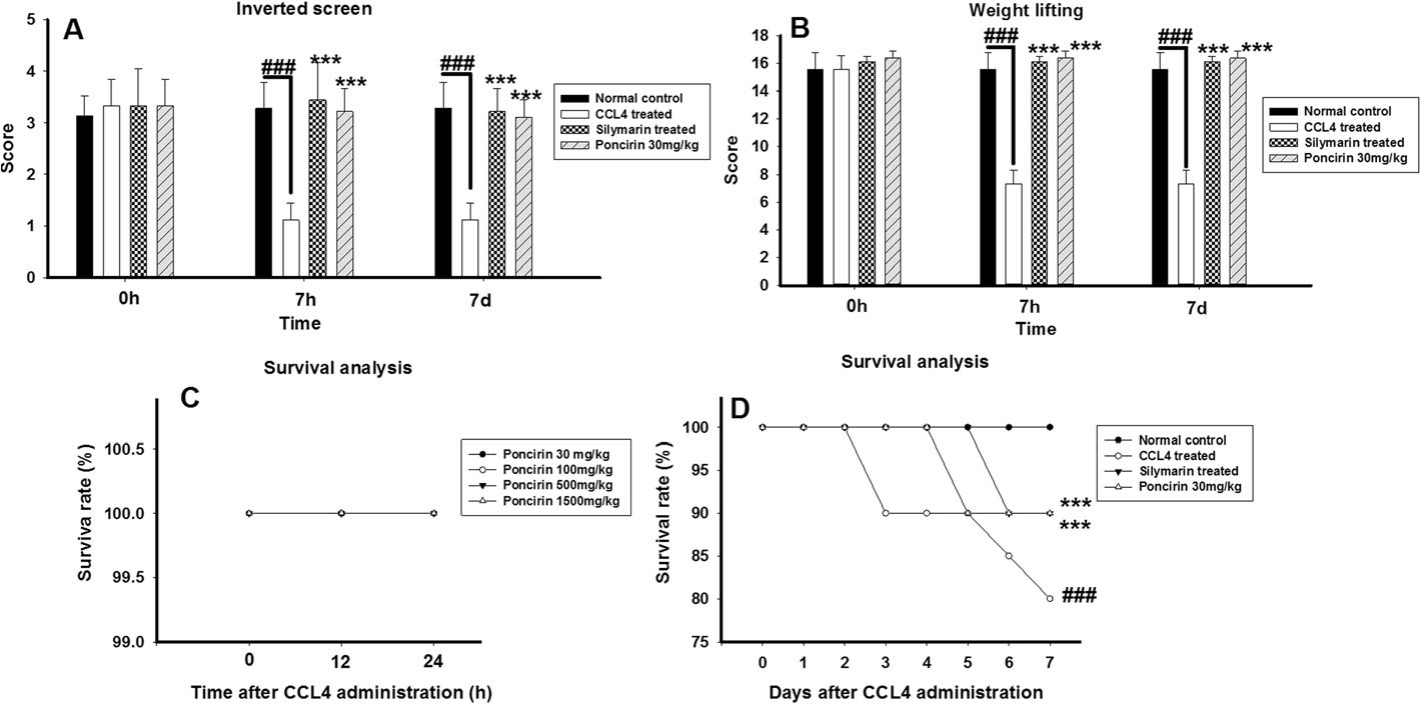
The effect of the poncirin on the locomotor and survival rate (acute and chronic) in CCL4-induced liver injury. The poncirin treatment did not significantly altered the locomotor activity such as inverted screen (**a**), weight lifting (**b**) and showed marked survival rate (**c**, **d**) compared to the CCL4 treated group. The silymarin treated group also markedly improved the locomotor activity and survival rate. The data was reported as mean ± standard deviation (*) *p* < 0.05, (**) *p* < 0.01, (***) *p* < 0.001, and (###) shows significant difference comparison with CCL4 treated group

The muscle strength and coordination analysis showed that the poncirin treatment did not hampered the locomotor functions. However, the CCL4-induced group showed marked alteration in the body locomotor activity using inverted screen mesh and weight chain. Furthermore, the silymarin treated animals also did not interfere with the muscle strength and coordination as shown in Fig. 6.

### Survival analysis

The effect of the poncirin treatment on the survival rate was studied after the CCL4 administration. The poncirin treatment significantly decrease the mortality rate compared to the CCL4 treated groups. Similarly, the silymarin also obviously improved the survival rate and decreased the mortality rate in contrast to the negative control as shown in Fig. 6.

### Effect of poncirin treatment on the blood chemistry

The hematological tools can be employed to assess the diagnosis and prognosis of an inflammatory disease like hepatotoxicity. The xenobiotics like CCL4 can induce eryptosis i.e. the destruction of aged and damaged red blood cells, causing increased degradation of RBCs from the systemic circulation and associated with the symptoms of anemia [28]. Similarly, the increased degradation of RBCs also causes the decrease in the hemoglobin. Furthermore, the inflammatory conditions are associated with the increased in white blood cells count. However, the poncirin treatment improved the RBCs and hemoglobin concentration and brought WBCs count down. Similarly, the CCL4 also decreased the platelets count, however, poncirin treatment improved the platelet count as shown in the Table 1.

**Table 1:** Effect of treatment with poncirin on hematology profile

Parameters	WBC (10^**9**^/L)	LYM (10^**9**^/L)	NEU (10^**9**^/L)	MON (10^**9**^/L)	EOS (10^**9**^/L)	PLT (10^**9**^/L)	RBC (10^**12**^/L)	HB (g/dL)
**Normal control**	3.91 ± 0.30	4.2 ± 0.90	7.1 ± 0.04	0.4 ± 0.10	0.21 ± 0.05	213.0 ± 15.01	13.1 ± 22	15.3 ± 0.34
**CCl4 Treated**	7.01 ± 0.32###	6.3 ± 0.30###	11.2 ± 0.3###	0.91 ± 0.30###	0.40 ± 0.01###	423.7 ± 9.0###	8.5 ± 13.6###	11.5 ± 1.0###
**Silymarin 50 mg/kg**	4.56 ± 0.11**	4.7 ± 0.21*	7.6 ± 0.21*	0.53 ± 0.20*	0.24 ± 0.20**	278.2 ± 3.2**	12.3 ± 25*	14.0 ± 0.2*
**Poncirin 30 mg/kg**	4.89 ± 0.23**	4.8 ± 0.02*	8.2 ± 0.09*	0.50 ± 0.12*	0.27 ± 0.02**	256.0 ± 87**	12.0 ± 21*	14.3 ± 0.45*

All values are expressed as mean ± SEM (n = 8). ^###^*P* < 0.05 compared with vehicle control group^*^P < 0.05, **P < 0.01 and ***P < 0.001 compared with negative control group

### Effect of poncirin on biochemical parameters and serum enzymes

The effect of the poncirin on the CCL4-induced liver injury markers such as ALT, AST, ALP, total protein and bilirubin were evaluated. The poncirin treatment significantly reduced the liver stress markers such as bilirubin, ALT, ALP, AST and enhanced the serum concentration of total protein as evident from the Table 2**.**

**Table 2:** Effect of treatment with poncirin on biochemical parameters

Sample	ALT (μl/dl)	AST (μl/dl)	ALP (μ/dl)	Total Bilirubin (mg/dl)	Total Protein (g/dl)
**Normal control**	43.12 ± 2.5	40.03 ± 0.7	37.04 ± 1.8	0.24 ± .03	8.18 ± 0.36
**CCL4 treated**	215.1 ± 1.3^###^	198.1 ± 3.0^###^	66.3 ± 2.6^###^	1.04 ± 0.06^###^	4.44 ± 0.46^###^
**Silymarin 50 mg/kg**	78.8 ± 4.24**	84.5 ± 5.65*	40.23 ± 1.4**	0.375 ± 0.10**	7.52 ± 0.14*
**Poncirin 30 mg/kg**	80.3 ± 4.04**	95.6 ± 8.14*	40.65 ± 2.8**	0.38 ± 0.22**	7.89 ± 0.31*

All values are expressed as mean ± SEM (*n* = 8). ^###^*P* < 0.05 compared with vehicle control group^*^P < 0.05, **P < 0.01 and ***P < 0.001 compared with negative control group

### Effect of the poncirin treatment on the CCL4-induced electrolytes imbalance

The CCL4 administration was observed for the severe alteration in the electrolytes profile of the serum such as sodium, potassium and bicarbonate [28]. The level of the sodium and potassium were markedly reduced following CCL4 administration. Furthermore, the level of the bicarbonate was strikingly lowered by the CCL4 mediated liver injury. However, the therapeutic intervention of poncirin significantly improved the level of the sodium, potassium and bicarbonate as depicted from the Table 3.

**Table 3:** Effect of treatment with poncirin on electrolyte profile

Sample	Sodium (mEq/L)	Potassium (mEq/L)	Bicarbonate (mEq/L)
**Normal control**	132.1 ± 0.01	9.5 ± 0.7	27.5 ± 0.4
**CCL4 treated**	111.6 ± 0.70^###^	10.3 ± 0.56^###^	38.4 ± 1.7^###^
**Silymarin 50 mg/kg**	124.03 ± 0.3***	9.3 ± 0.78	31.09 ± 0.06**
**Poncirin 30 mg/kg**	120.24 ± 0.50***	8.43 ± 0.30	32.9 ± 0.09*

All values are expressed as mean ± SEM (*n* = 8). ^###^*P* < 0.05 compared with vehicle control group^*^P < 0.05, **P < 0.01 and ***P < 0.001 compared with negative control group

### Effect of the poncirin on the CCL4-induced NO production in plasma and liver

**Fig. 7:**
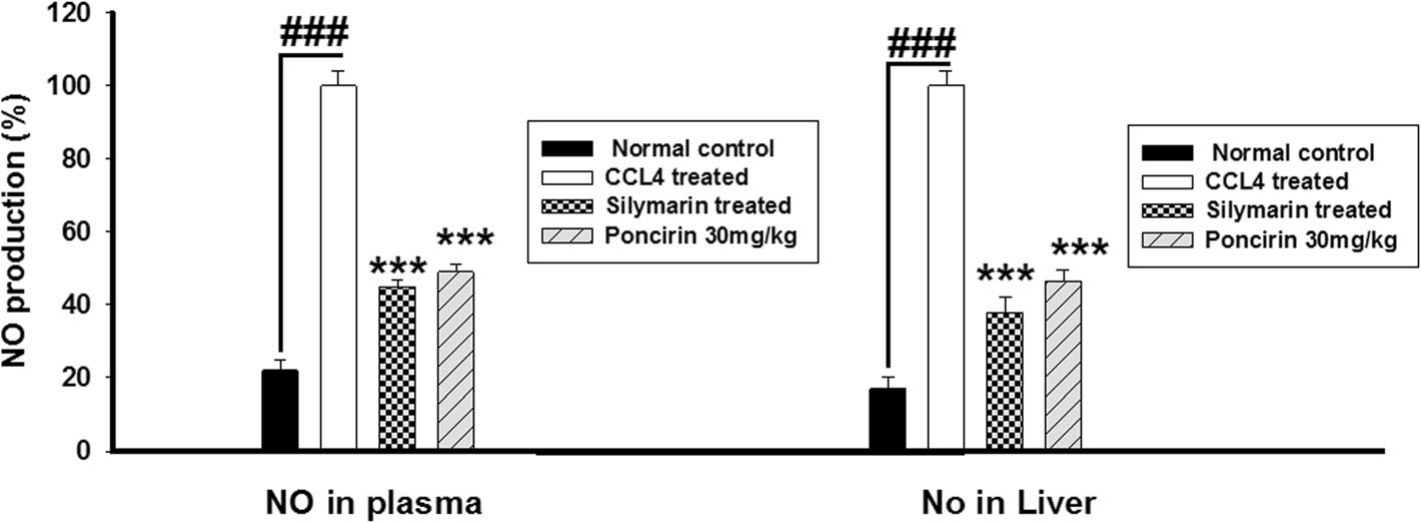
The effect of poncirin treatment on the NO production in plasma and liver tissue in CCL4-induced liver injury model. The CCL4 treatment significantly increased the NO production in both plasma (**a**) and liver tissue (**b**). The poncirin treatment significantly attenuated the NO production in both plasma and liver tissue. Similarly, the silymarin treatment also promisingly reduced the production of the NO compared to the CCL4 treated group. The data was reported as mean ± standard deviation (*) *p* < 0.05, (**) *p* < 0.01, (***) *p* < 0.001, and (###) shows significant difference comparison with CCL4 treated group

The effect of poncirin treatment was evaluated on the NO production in both plasma and liver tissue homogenate using Griess reagent method [38]. The poncirin treatment significantly neutralized the NO production and hence, inflammatory process following liver injury. The silymarin treated group also exhibited marked reduction in the NO production in both plasma and liver tissue at day 7 of the CCL4 administration Fig. 7.

### The poncirin treatment improve histological parameters

**Fig. 8:**
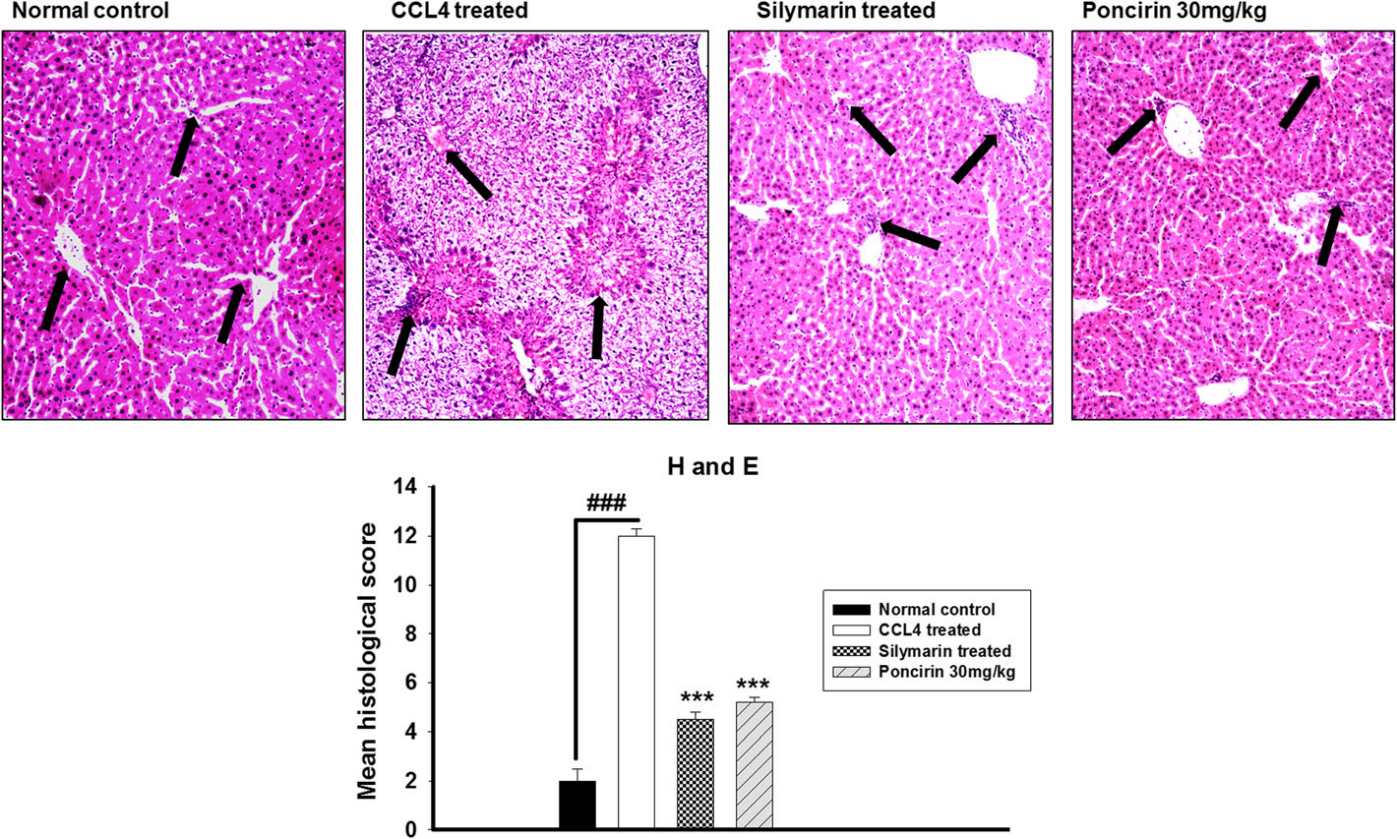
The effect of the poncirin treatment on histological changes of the liver in CCL4-induced liver injury model. The H and E staining was employed to assess the hepatic inflammation, Centrilobular necrosis, cellular hypertrophy and vacuolization. The CCL4 treated group marked alteration in histology and showed marked inflammation. However, the poncirin treatment significantly improved the histology compared to the CCL4 treated group and the relative histological score was plotted. The data was reported as mean ± standard deviation (*) *p* < 0.05, (**) *p* < 0.01, (***) *p* < 0.001, and (###) shows significant difference comparison with CCL4 treated group

The Hemotoxylin and Eosin staining was performed to assess the CCL4 mediated liver damage and determine the effect of the poncirin treatment on the liver histology. The H and E staining showed that poncirin treatment significantly improved the histological parameters in contrast to the control. Similarly, the positive control also improved the histopathology of the liver as shown Fig. 8.

### Effect of poncirin administration on the antioxidant mechanism such as GSH, GST, catalase and SOD

**Fig. 9:**
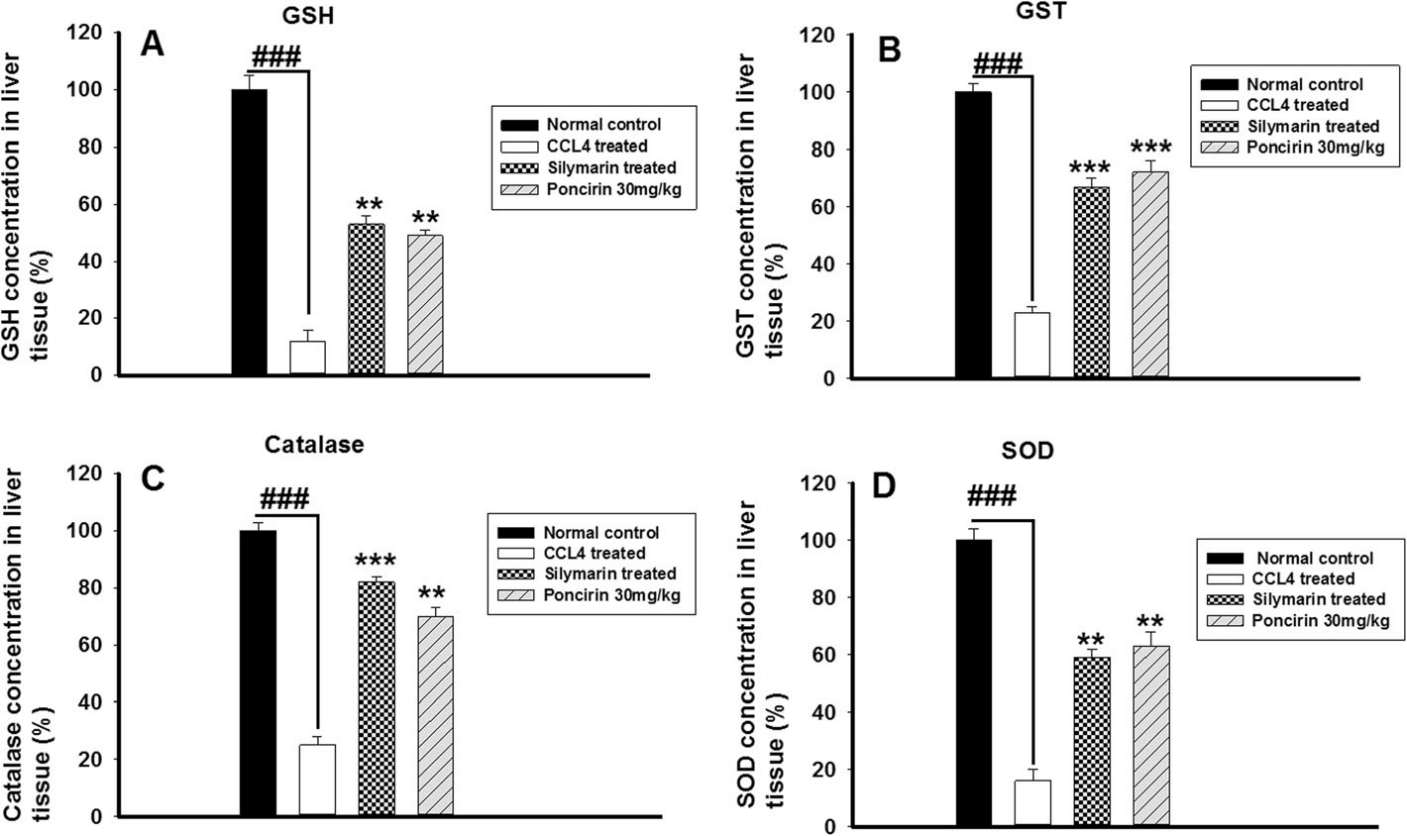
The effect of the poncirin treatment on the anti-oxidants enzymes such as GSH (**a**), GST (**b**), Catalase (**c**) and SOD (D) in CCL4-induced liver injury. The poncirin treatment significantly induced the anti-oxidant enzymes compared to the CCL4 treated group. The silymarin treated group also enhanced the anti-oxidant enzymes compared to the CCL4 treated group. The data was reported as mean ± standard deviation (*) *p* < 0.05, (**) *p* < 0.01, (***) *p* < 0.001, and (###) shows significant difference comparison with CCL4 treated group

The poncirin treatment significantly enhanced the antioxidant mechanism such as GSH, GST, Catalase, SOD and thus, protect the hepatocyte from the CCL4-induced oxidative stress. The positive control treated with the silymarin also induced the antioxidant mechanism to counteract the oxidative stress posed by the CCL4 as shown in Fig. 9.

### Effect of poncirin on LPO and MPO activity in CCL4-induced liver injury model

**Fig. 10:**
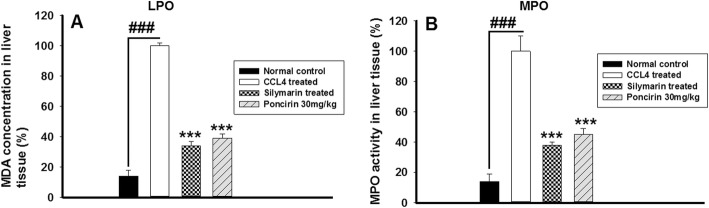
The effect of the poncirin treatment on the CCL4–induced changes in LPO and MPO activity in liver. The poncirin treatment markedly attenuated the LPO (A) and (MPO) activity within the liver tissue induced by the CCL4 administration. The silymarin treated group also improved the LPO and MPO activity within the liver tissue compared to the CCL4 treated group

The lipid peroxidase assay was performed to assess the extent of lipid peroxidation and subsequently, to assess the activity of the poncirin against the oxidative stress induced by the CCL4 [39, 40]. The CCL4 administration was associated with pronounced increase in the LPO activity, however, the poncirin treatment showed striking decrease in the LPO activity as evident from Fig. 10a. Additionally, the MPO assay was performed to assess the infiltration of the neutrophils into the tissue and is used as the marker of the neutrophilic infiltration into the tissue. The MPO assay was performed using HTAB and o-dianisidine as reported previously [39, 40]. The poncirin treatment significantly inhibited the MPO activity compared to the CCL4 treated group. The silymarin treated group also markedly attenuated the MPO activity compared to the CCL4 treated group Fig. 10b.

### The poncirin treatment inhibited the Evans blue vascular permeability

**Fig. 11:**
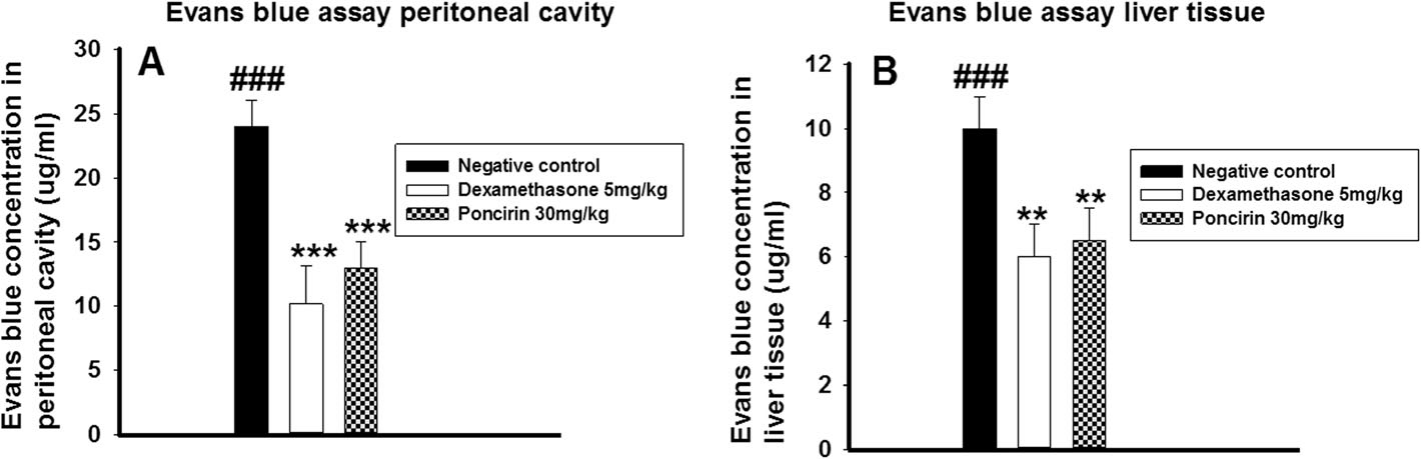
The effect of the poncirin treatment on acetic acid-induced Evans blue vascular permeability. The acetic acid treated group showed marked permeation of the Evans blue into the peritoneum (**a**) and liver tissue (**b**). However, the dexamethasone and the poncirin treated group showed marked decrease in the Evans blue permeability compared to the CCL4 treated group. The data was reported as mean ± standard deviation (*) *p* < 0.05, (**) *p* < 0.01, (***) *p* < 0.001, and (###) shows significant difference comparison with CCL4 treated group

The acetic acid administration significantly enhanced the Evans blue permeability into peritoneum and liver tissue compared to negative control group. However, poncirin treatment dose dependently altered the Evans blue vascular distribution into the liver tissue, brain tissue as well as in the peritoneal cavity in contrast to the control as depicted in the figure. The dexamethasone treated group also shared similar results and markedly inhibited the Evans blue vascular permeability in CCL4-induced hepatotoxicity as shown in Fig. 11.

### Effect of poncirin on the pro-inflammatory mediators in liver after CCL4 administration

**Fig. 12:**
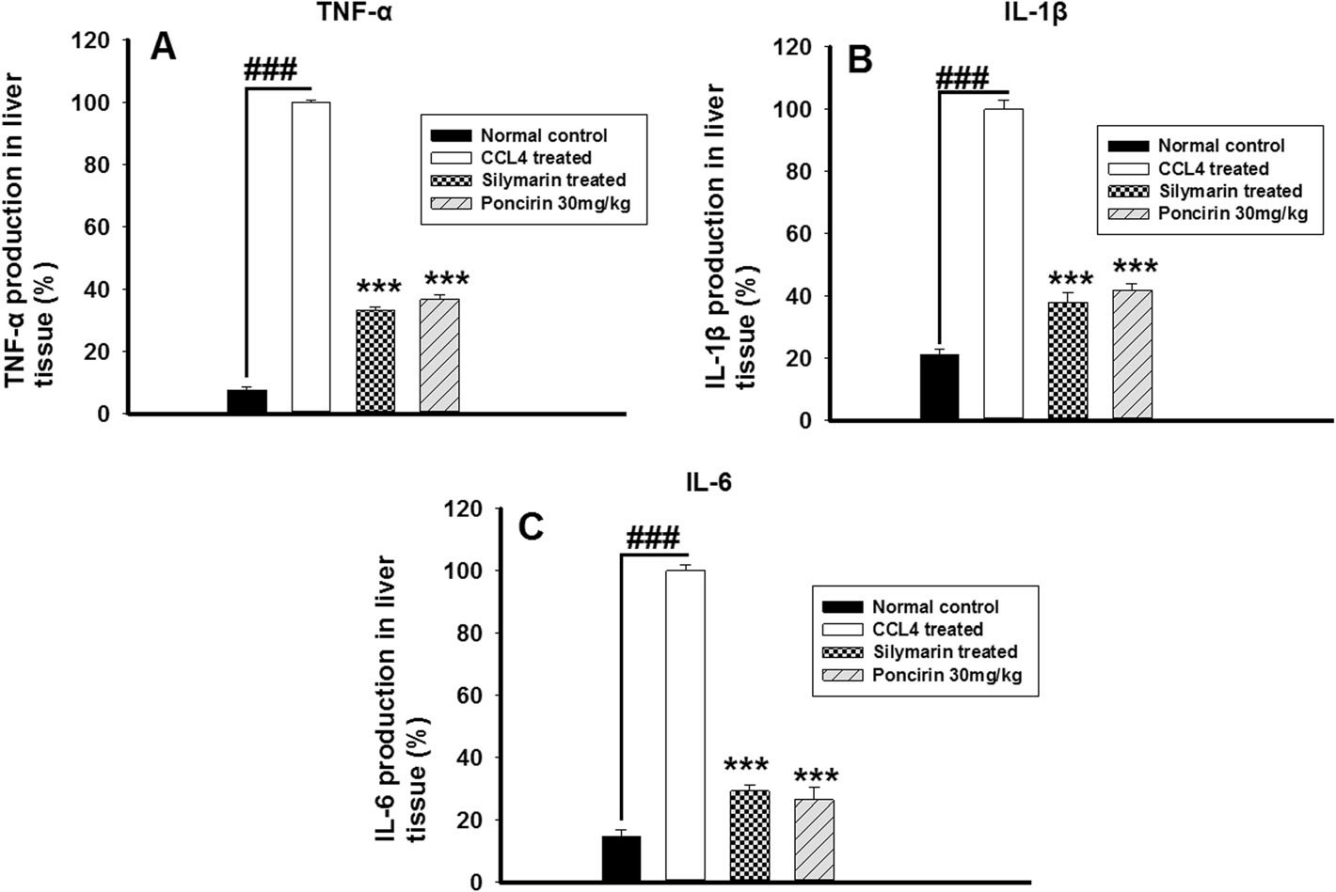
The effect of the poncirin treatment on the TNF-α (**a**), IL-1β (**b**) and IL-6 in CCL4-induced liver injury model. The CCL4 administration significantly enhanced the production of the pro-inflammatory cytokines such as TNF-α, IL-1β and IL-6. However, the poncirin treatment markedly attenuated the production of the pro-inflammatory cytokines. The silymarin treatment also markedly reduced production of the inflammatory cytokines. The data was reported as mean ± standard deviation (*) *p* < 0.05, (**) *p* < 0.01, (***) *p* < 0.001, and (###) shows significant difference comparison with CCL4 treated group

The effect of the poncirin treatment was assessed on the production of pro-inflammatory mediators (IL-1β, IL-6 and TNF-α) after induction of liver injury with the CCL4. The poncirin treatment markedly attenuated the production of these stated cytokines using Elisa assay compared to negative control group at day 7. Similarly, the same effect was observed with the silymarin treated group as depicted Fig. 12.

### Molecular docking study

**Fig. 13:**
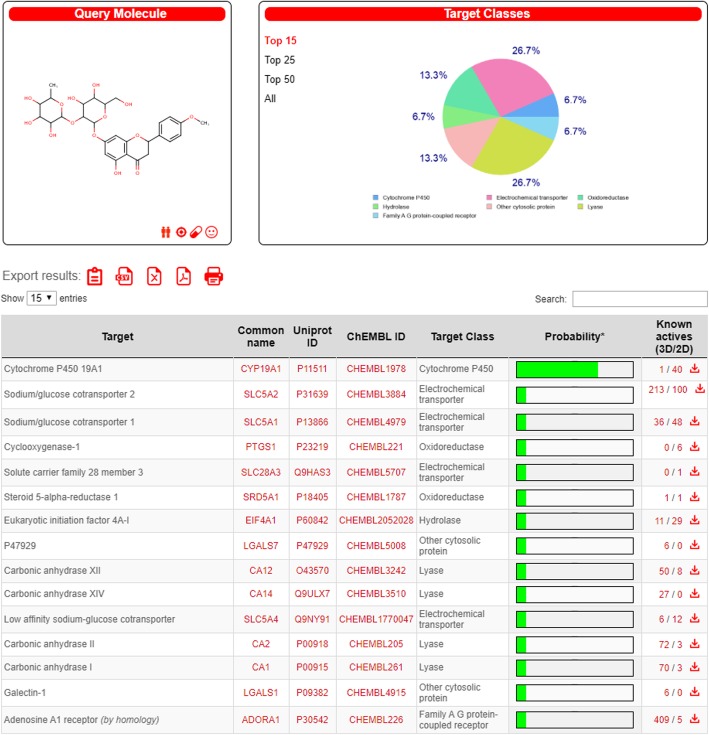
The effect of the poncirin with the various targets were studied using Swiss Target Prediction version-2019. The poncirin showed significant interaction with the cytochrome p450, while good interaction with the other targets

The poncirin showed significant interaction with the cytochrome p45019A1 using Swiss Target Prediction version-2019. These enzymes are actively involved in the metabolism and encounter the oxidative stress marker. Furthermore, the poncirin also showed interaction with various target proteins Fig. 13.

## Discussion

The liver is the vital organ of the body and part of GIT associated with the regulation of numerous functions of the body including metabolism, synthetics and detoxification [41, 42]. The CCL4 is well known toxicants associated with the liver damage by producing the oxidative stress and damage of the cellular components [43]. In the current study, the CCL4-induced models was used to assess the anti-inflammatory and hepatoprotective activity of the poncirin in animal models. The effect of poncirin was investigated against both acute and chronic CCL4 model [44]. The poncirin treatment significantly improved the physiological parameters such as food and water intake compared to the CCL4 treated group, which showed less food and water intake. Change in body weight are associated with the chronic inflammatory disease conditions and it might be due to less intake of food. The CCL4 administration marginally reduced the body weight, while the positive control and poncirin treated group exhibited no such decrease in the body weights compared. During inflammatory conditions the organs become heavy and boggy compared to the normal organs. In the current study, the liver weight assessment was performed to assess the changes in liver weights in all the recruited groups. The CCL4-induced group revealed marked increase in the liver weight compared, however, the positive control treated with the silymarin and poncirin showed marginal decrease in the liver weight.

The hematology study showed marked alteration in the blood composition and decrease RBCs, HB content, while raised level of WBCs was noticed in the CCL4 treated group. However, the poncirin treatment improved the hematological parameters and RBCs, HB content was improved, while significant reduction in the WBCs was noted. The ALT, AST, ALP and bilirubin are employed clinically to assess the hepatocytes destruction, while the total protein is widely used to assess the synthetic activity of the liver [45]. The study showed that CCL4 groups was associated with significant increase in the ALT, AST, ALP and creatinine, while lowered level of the total protein concentration was noted. However, the poncirin treated group showed significant decrease in the hepatocytes damaging parameters and revealed preserved synthetic activity. The electrolytes imbalances was noticed in the CCL4-induced liver injury and the level of potassium, sodium and bicarbonate were altered. However, poncirin treatment reversed the CCL4 mediated electrolytes changes compared to the negative control. The various inflammatory conditions are associated with enhanced nociceptive responses and decrease the pain threshold [46]. Acetic acid administration significantly elevated the Evans blue vascular permeability into peritoneum and liver tissue [46, 47]. However, the poncirin treatment markedly inhibited the Evans blue leakage into the vascular and tissue compartment in contrast to negative control [46, 47]. In the present study, the H and E staining was performed to assess the effect of poncirin treatment on the CCL4-induced liver injury [48]. The only CCL4 treated groups exhibited significant disturbance liver tissue, however, the poncirin treatment markedly improved the histological parameters [48]. Furthermore, the CCL4 administration significantly enhanced the oxidative stress and the concentration of the MDA, which is the end product of the lipid peroxidation was markedly enhanced [49]. However, the poncirin treatment significantly attenuated the production of the MDA and hence, inhibited the oxidative stress in contrast to the negative control [49, 50].

Additionally, the MPO activity served as a marker for the neutrophilic infiltration and indicates the degree of the inflammation. The CCL4 treated group showed marked increase in the MPO activity [51]. The antioxidant mechanism such as GST, GSH, Catalase and SOD were markedly compromised by the CCL4 administration, while the poncirin treatment strikingly reversed the antioxidant mechanism as compared to the only CCL4 treated groups [51]. The effect of the poncirin on the CCL4 mediated NO production was investigated and it was observed that the poncirin treatment significantly altered the NO production in contrast to the only CCL4 administered group [52]. The inflammatory cytokines plays key role in almost all disease including liver injury [44]. The CCL4 administration was observed with raise in the level of pro-inflammatory mediators such as IL-1β, IL-6 and TNF-α. However, the poncirin treatment showed marked reduction in the inflammatory cytokines compared to the negative control.

## Conclusions

In conclusion the poncirin administration showed marked hepatoprotection against the acute and chronic CCL4-induced liver injury model. The poncirin significantly improved the hematological, biochemical and histological parameters, while attenuated the inflammatory and oxidative stress markers. Furthermore, the poncirin treatment exhibited no observable toxicity against the animals. Thus, based on the current study the poncirin can prove potential candidate against the liver injury.

## Data Availability

The data presented in this study are confined and described within the article and will be disseminated by the corresponding author upon the reasonable request. All materials used in this study are included in Methods section adequately.

## Abbreviations

CCL4: Carbon tetrachloride
IL: Interleukin
NO: Nitric oxide
MDA: Malonaldehde
TNF-α: Tumor necrosis factor-α
MPO: Myeloperoxidase
GIT: Gastrointestinal tract
SOD: Sulphuroxide dismutase
ALT: Alanine transaminase
AST: Aspartate aminotransferase
ALP: Alkaline phosphatase
WBCs: White blood cells
RBCs: Red blood cells
H and E staining: Hematoxylin and eosin staining
HTAB: Hexadecyltrimethylammonium bromide
LPO: Lipid peroxidase
iNOS: Inducible nitric oxide synthase
MAPKs: Mitogen activated protein kinases
MyD88: Myeloid differentiation88
NF-κB: Nuclear factor kappa-light-chain-enhancer of activated B cells
IRAK: Interleukin-1 receptor associated kinase
MPO: Myeloperoxidase
CCL4: Carbon tetra chloride
